# Slow pseudo sinus rhythm and atrial tachycardia of right superior venous pulmonary origin

**DOI:** 10.1093/omcr/omab093

**Published:** 2021-10-26

**Authors:** Thierry Verbeet, Thomas Nguyen, Alexandre Almorad, Maurice Jottrand, Thierry Wauters, José Castro

**Affiliations:** 1 Department of Cardiology, University Hospital Brugmann, 1020 Brussels, Belgium; 2 Department of Cardiology, La Médicale Montgomery, 1150 Brussels, Belgium

**Keywords:** cardiac electrophysiology

## Abstract

Paroxysmal atrial tachycardia usually presents as a sudden acceleration of the atrial rate combined with modifications of the P wave morphology. A 22-year-old patient presented with very fast and very slow atrial ectopic activity. He complained of repetitive episodes of fast tachycardia, some accompanied with dizziness.

When the ectopic discharge was slow, no clear-cut difference between the sinus rate and the ectopic rate was seen and thus the atrial rhythm appeared quite regular. The ectopic focus was situated deep inside the right upper pulmonary vein (RSPV).

After RSPV isolation a persistent sinus rhythm was established and since then the patient has been asymptomatic for 3 years.

Thus, subtle changes in the P wave morphology without a significant change in the heart rate in patients presenting with palpitations can give a clue to the diagnosis of the tachycardia and the localization of the ectopic focus.

## INTRODUCTION

Subtle changes in the P wave polarity combined with slight changes in the heart rate are often considered to represent a shift of the origin of the impulse within the sinus node itself.

## CASE REPORT

A 22-year-old man was referred to us for drug resistant atrial tachycardia during exercise since 4–5 years. The rate of the tachycardia had been measured up to 250 bpm inducing marked dizziness. Slower ectopic rhythms around 100 bpm were also recorded. Various degrees of atrioventricular block could also be seen during tachycardia. The patient was initially treated with flecainide 100 mg bid but since 3–4 months the tachycardia recurred many times a week lasting from 1 minute to a couple of hours. There was no past medical history. Physical examination was normal. The echocardiogram was normal, too.

An electrophysiological study was performed.

**Figure 1:**
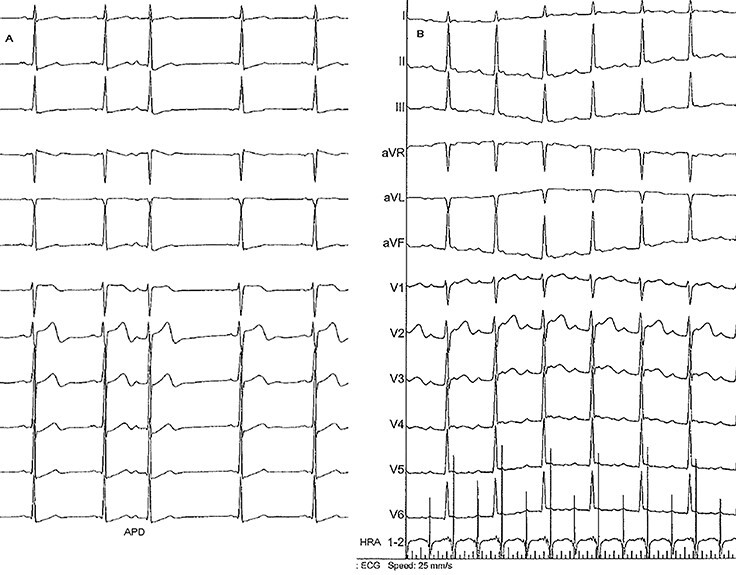
(A) One atrial premature beat (APD) shows a slight reduction of the positive P wave amplitude in Lead 1, increase of the positivity in Lead 1, Lead 3, AVF, negativation of the P wave in AVL and positivation in V1 compared with sinus rhythm. (B) Fast atrial tachycardia with 2/1 atrioventricular block demonstrating the presence of an atrial tachycardia. The atrial rate is 220 bpm. The P wave polarity is similar to that of the atrial premature beats.

Initially, only supraventricular premature beats were seen. Compared with sinus rhythm, there was a slight reduction in the amplitude of the positive P in Lead 1, the P waves were more positive in Lead 2, Lead 3, AVF, the P waves were inverted in AVL compared with sinus rhythm and there was a change in polarity of the P wave in V1 from biphasic positive negative to positive (Fig. 1A).

A non-sustained atrial tachycardia at 220 bpm with 2/1 block occurred after atrial burst pacing and showed P waves of the same polarity as the premature beats (Fig. 1B).

**Figure 2:**
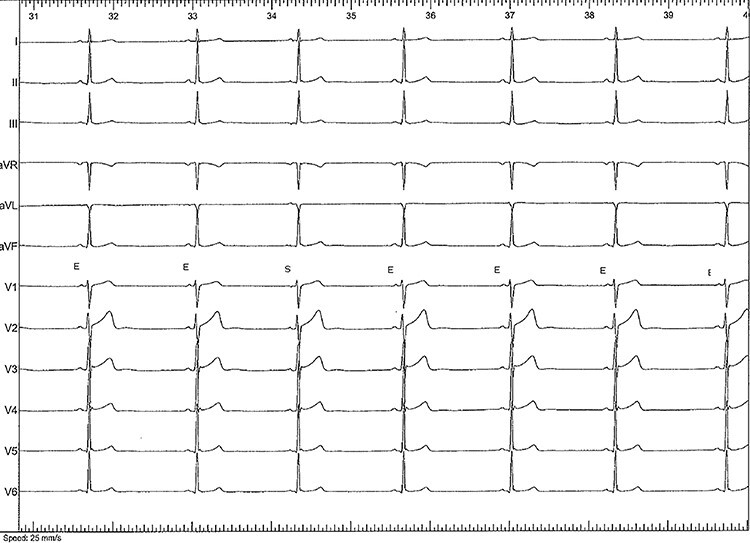
Slow regular atrial rhythm around 45 bpm. Two morphologies of P waves can be seen. One (E) is similar to the ectopic rhythm recorded during the fast tachycardia and the premature atrial beats. One sinus beat can also be seen (S).

At times, a slow regular atrial rhythm around 45 bpm was seen with alternating sinus and ectopic rhythm (Fig. 2).

Intracardiac measurements showed that during the premature beats and the tachycardia the high right atrium and the coronary sinus os were activated simultaneously and that the lateral wall of the right atrium was late.

At that point of time, the differential diagnosis based on right atrial mapping was: a focus in the right interatrial septum, a focus in the non-coronary aortic cusp, a focus in the left interatrial septum, a focus in the right upper pulmonary vein. The earliest atrial activation during the arrhythmia was found in the high right septal area with a prematurity of minus 30 ms in relation with the beginning of the P wave. Pacing at that site was the best site to reproduce closely the timing and the morphology of the right intra atrial and coronary sinus electrograms during the arrhythmia.

One radiofrequency application at that site was unsuccessful. The left atrium was accessed via transseptal puncture. A Biosense Lasso catheter was positioned in the right superior pulmonary vein (RPSV) as well as a Biosense Thermocool Smarttouch D curve ablation catheter. These catheters were connected to a 3D electroanatomical system (Carto3, Biosense Webster, USA).

**Figure 3:**
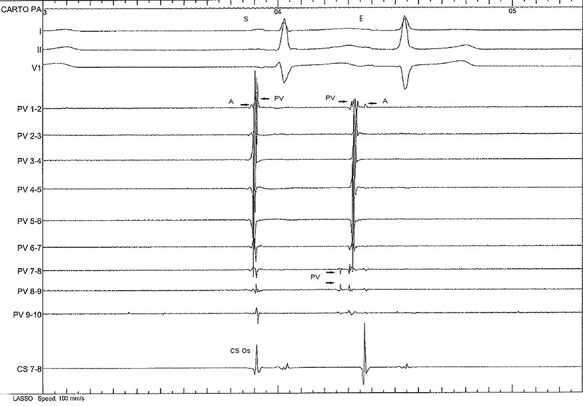
The Lasso catheter (PV1–2 = > 9–10) is positioned at the ostium of the right superior pulmonary vein. PV 1–2 records the roof of the vein. One sinus beat (S) followed by one ectopic beat (E) is seen. The Lasso recording shows a normal sequence of activation on the sinus beat: a far field atrial activity (A) followed by a pulmonary vein potential (PV). The ectopic beat shows an inversion of the sequence of activation (PV precedes A) with fragmented PV potentials (PV 7–10) preceding the far field atrial potentials (A) and the beginning of the P wave by 100 ms and also preceding the other PV potentials recorded by the Lasso catheter demonstrating the pulmonary venous origin of the ectopy.

After placement of the Lasso in the RSPV, at the antero–inferior part of the Lasso (electrodes 7–10), small fragmented potentials with a prematurity of minus 100 ms in relation with the beginning of the P wave during extrasystoles were seen. There was also an inversion of the normal sequence of RPSV activation: the venous potential preceded the far field atrial potential on the premature beats (Fig. 3), demonstrating the pulmonary venous origin.

**Figure 4:**
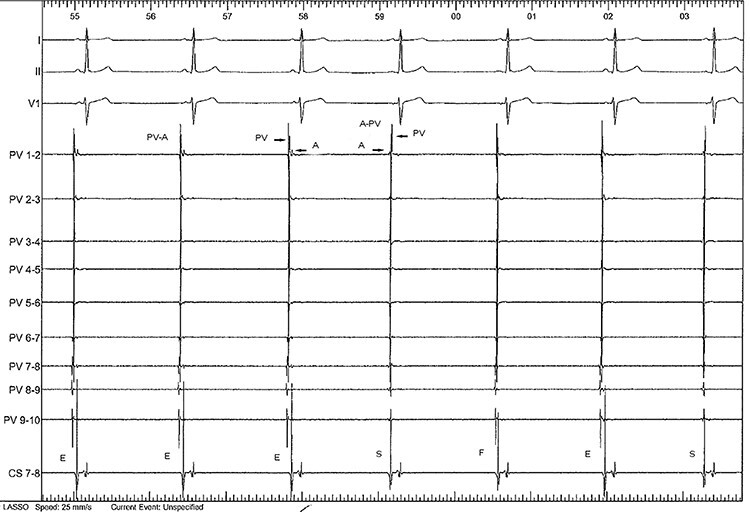
A regular atrial rhythm at 43 bpm is seen. The first three beat are ectopic (E), the fourth is of sinus origin (S), the fifth represents fusion between sinus and ectopic (F) followed by an ectopic beat and a sinus beat. There are only slight atrial cycle length changes during this sequence. (A) represents the far field atrial potential. PV represents the RSPV pulmonary venous potential. On Lasso PV 1–2 the inversion of the normal sinus sequence of activation (A-PV) can be seen during the ectopy (PV-A). This change is magnified at 100 mm/s on Fig. 3.

We could also see an alternation of sinus rhythm and RPSV regular atrial rhythm around 43 bpm with inversion of the normal sequence of activation of the pulmonary vein on the abnormal beats (Fig. 4).

The RPSV was isolated with an encircling lesion. After isolation was completed, a slow irregular dissociated rhythm with a cycle length between 380 and 680 ms was visible inside the vein. Mapping the dissociated rhythm within the RSPV with the ablation catheter showed fragmented potentials preceding the earliest Lasso activation by 35 ms. These were recorded a few centimeters inside the vein, beyond the right heart radiologic border.

During a 24 hour follow-up only the typical sinus node atrial activation could be seen.

The patient remains asymptomatic after a follow-up of 3 years.

## DISCUSSION

This case report demonstrates that ectopy coming from the RPSV may cause rapid tachycardia but may also be responsible for very slow stable atrial ectopic atrial rhythm mimicking sinus rhythm. Pulmonary vein tachycardia has been shown to be associated with slow and fast ventricular rhythms in a limited number of cases. In some cases, it is, as in our case, due to slow and fast firing of the ectopic focus (3 cases described) [1–3]. In two of them however the focus was situated in the left superior pulmonary vein. In some cases permanent rapid pulmonary venous firing mimicking sinus tachycardia caused heart failure [4, 5].

As far as the polarity of the ectopic P wave is concerned, it was in accordance with the RSPV localization suggested by Yamane *et al.* in their study on pulmonary venous pacing [6].

Kistler *et al.* found in 27 cases that in the case of pulmonary venous ectopy 39% of the ectopic rhythms were coming from the RSPV and 39% from the left superior pulmonary vein. They also found that 93% of the pulmonary venous foci were ostial [7]. This was not the case in our patient where the focus was situated inside the vein.

The main take home message is the following: changes in the polarity of the P waves, even subtle, despite a slow regular atrial rhythm should raise attention in patients complaining of paroxysmal tachycardia and could be a clue to the presence of an ectopic focus responsible for the complaints.

The polarity of the P wave may help in localizing the origin of the focus.

This also illustrates the ability of the RPSV to generate very slow or very fast ectopic rhythms in the same patient.

## References

[1] Tao S, Yamauchi Y, Okada H, Maeda S, Naito T, Kagiyama N, et al. Pulmonary vein rhythm with prominent change in heart rate. Shinzo 2012;44:197–202.

[2] Yamane T, Shah DC, Jais P, Haïssaguerre M. Pseudo sinus rhythm originating from the left superior pulmonary vein in a patient with paroxysmal atrial fibrillation. J Cardiovasc Electrophysiol 2001;12:1190–1. 11699531 10.1046/j.1540-8167.2001.01190.x

[3] Mahajan R, Lim HS, Lau DH, Sanders P. Left superior pulmonary vein rhythm masquerading as sinus rhythm. Indian Pacing Electrophysiol J 2011;11:20–3. 21468275 PMC3065745

[4] Matsuo S, Yamane T, Date T, Mochizuki S. Pseudosinus tachycardias originating from left pulmonary veins. J Cardiovasc Electrophysiol 2006;17:682–4. 16836723 10.1111/j.1540-8167.2006.00356.x

[5] Scavee C, Brasseur A, Weerasooriya R. A pseudo sinus tachycardia originating from the right superior pulmonary vein. Successful ablation by a simplified, targeted ablation strategy. Acta Cardiol 2018;63:265–9. 10.2143/AC.63.2.2029537 18468209

[6] Yamane T, Shah DC, Peng JT, Jaïs P, Hocini M, Deisenhofer I, et al. Morphological characteristics of P waves during selective pulmonary vein pacing. J Am Coll Cardiol 2001;38:1505–10. 11691531 10.1016/s0735-1097(01)01578-9

[7] Kistler PM, Sanders P, Fynn SP, Stevenson IH, Hussin A, Vohra JK, et al. Electrophysiological and electrocardiographic characteristics of focal atrial tachycardia originating from the pulmonary veins: acute and long-term outcomes of radiofrequency ablation. Circulation 2003;108:1968–75. 14557361 10.1161/01.CIR.0000095269.36984.75

